# The Transcriptional Repressor Protein NsrR Senses Nitric Oxide Directly via a [2Fe-2S] Cluster

**DOI:** 10.1371/journal.pone.0003623

**Published:** 2008-11-07

**Authors:** Nicholas P. Tucker, Matthew G. Hicks, Thomas A. Clarke, Jason C. Crack, Govind Chandra, Nick E. Le Brun, Ray Dixon, Matthew I. Hutchings

**Affiliations:** 1 Department of Molecular Microbiology, John Innes Centre, Norwich Research Park, Norwich, United Kingdom; 2 School of Biological Sciences, University of East Anglia, Norwich Research Park, Norwich, United Kingdom; 3 School of Chemical Sciences and Pharmacy, University of East Anglia, Norwich Research Park, Norwich, United Kingdom; 4 School of Medicine, Health Policy and Practice, University of East Anglia, Norwich Research Park, Norwich, United Kingdom; Temasek Life Sciences Laboratory, Singapore

## Abstract

The regulatory protein NsrR, a member of the Rrf2 family of transcription repressors, is specifically dedicated to sensing nitric oxide (NO) in a variety of pathogenic and non-pathogenic bacteria. It has been proposed that NO directly modulates NsrR activity by interacting with a predicted [Fe-S] cluster in the NsrR protein, but no experimental evidence has been published to support this hypothesis. Here we report the purification of NsrR from the obligate aerobe *Streptomyces coelicolor*. We demonstrate using UV-visible, near UV CD and EPR spectroscopy that the protein contains an NO-sensitive [2Fe-2S] cluster when purified from *E. coli*. Upon exposure of NsrR to NO, the cluster is nitrosylated, which results in the loss of DNA binding activity as detected by bandshift assays. Removal of the [2Fe-2S] cluster to generate apo-NsrR also resulted in loss of DNA binding activity. This is the first demonstration that NsrR contains an NO-sensitive [2Fe-2S] cluster that is required for DNA binding activity.

## Introduction

Nitric oxide (NO) is a highly reactive and toxic free radical gas that can freely diffuse into cells and attack the redox centers of proteins. Human macrophages produce NO as a very early line of defense against invading bacterial pathogens. Soil bacteria are exposed to NO produced by denitrifying microbes and by the NO synthases of plants and microbes. Bacteria have evolved specific NO sensor proteins that regulate the expression of enzymes required for rapid detoxification of NO, usually by reduction to nitrous oxide (N_2_O), a greenhouse gas 300 times more potent than CO_2_ [1]. Understanding the ways in which bacteria sense and respond to NO is fundamentally important and has serious implications for human health, agriculture and the environment.

The Gram-negative bacterium *Escherichia coli* encodes several proteins that are known to sense NO directly, including the [4Fe-4S] oxygen sensing transcription factor FNR [2] and the [2Fe-2S] transcription factor SoxR [3]. However, two proteins appear to be dedicated solely to sensing NO in *E. coli*. The first is NorR, which senses NO directly through a mononuclear non-heme iron center [4] and responds by switching on expression of the flavorubredoxin NorVW to detoxify NO [5], [6], [7]. More recently a second protein, named NsrR [8], was shown to sense NO in *E. coli* [9] and to control a regulon of at least 30 genes [10]. This regulon includes *hmp*, which encodes an NO detoxifying flavohaemoglobin that converts NO to N_2_O or to nitrate (NO_3_^−^) [11]. NsrR, unlike NorR, appears to be a global regulator of NO-induced stress and has been identified and studied in a wide range of Gram-negative and Gram-positive bacteria including *Bacillus subtilis* [12], *Salmonella enterica* [13], and the obligate human pathogens *Neisseria meningitidis* [14] and *N. gonorrhoeae* [15]. NsrR belongs to the Rrf2 family [16] that includes the [2Fe-2S] containing transcription factor IscR and the iron regulator RirA [17], [18]. As a result it has been predicted that NsrR might also contain a [2Fe-2S] cluster that can sense NO directly.

**Figure 1:**
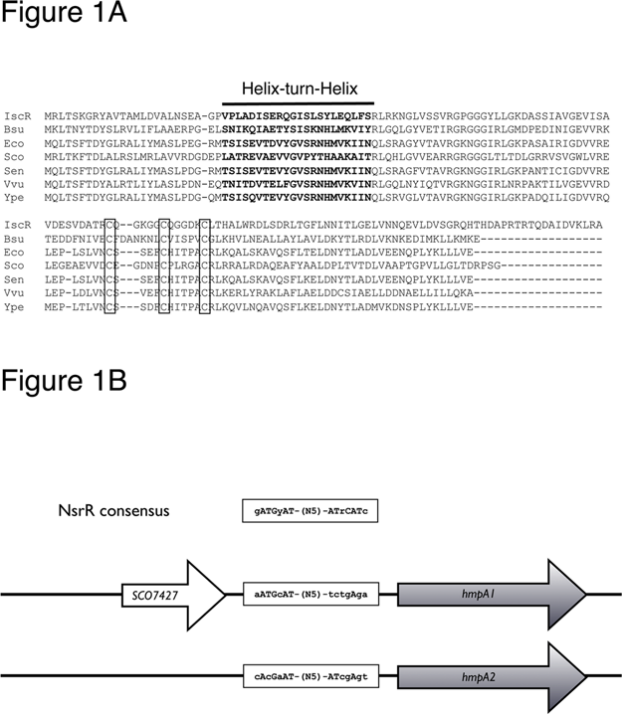
NsrR and the *hmpA* genes in *S. coelicolor*. (A). Alignment of *E. coli* IscR with NsrR sequences from *Bacillus subtilis* (Bsu), *E. coli* (Eco), *S. coelicolor* (Sco), *Salmonella enterica* Typhimurium (Sen), *Vibrio vulnificus* (Vvu) and *Yersinia pestis* (Ype). The helix-turn-helix DNA binding motif is highlighted. The three conserved cysteine residues predicted to ligate a [2Fe-2S] cluster are boxed. (B). *SCO7427* is the only gene encoding an Rrf2 family member in *S. coelicolor*. The coding sequence of *SCO7427* stops 74 base pairs upstream of the *hmpA1* start codon and is predicted to encode an NsrR homologue [16]. Both *hmpA* genes contain matches to the NsrR consensus for Bacillales and *Streptomyces* upstream of their translational start codons (boxes) [16].

NsrR homologues are encoded in many Gram-positive soil bacteria by genes that are usually linked to *hmp* [16]. To date, studies of NsrR have been restricted to the *in vivo* analysis of target genes in pathogenic bacteria such as *N. meningitidis* [14] or model organisms such as *E. coli* [9], [10] and *B. subtilis* [12]. Multiple sequence alignments revealed three conserved cysteine residues in the primary sequences of NsrR proteins (Fig. 1A) that could potentially act as ligands for a [2Fe-2S] cluster. Despite this, the presence of an NO-sensing metal center in NsrR has yet to be shown experimentally.

*Streptomyces* is a genus of ubiquitous saprophytic soil bacteria best known for producing useful secondary metabolites [19]. The model organism for the genus, *S. coelicolor*, an obligate aerobe, encodes two Hmp homologues (HmpA1 and HmpA2), which presumably detoxify NO by oxidising it to NO_3_^−^ or reducing it to N_2_O. The *hmpA1* gene is adjacent to a gene (*SCO7427*) encoding the only Rrf2 type protein encoded by the *S. coelicolor* genome (Fig. 1B). SCO7427 has previously been identified as an NsrR homologue using bioinformatics analysis [16] and alignment with other NsrR homologues shows that it contains the three conserved cysteine residues predicted to ligate the [2Fe-2S] cluster of NsrR (Fig. 1A). Here we report *in vitro* studies of *S. coelicolor* NsrR. We demonstrate that the protein can accommodate a [2Fe-2S] cluster, which is stable to atmospheric oxygen. We show that this form of the protein binds specifically to the promoter regions of *hmpA1* and *hmpA2*. The [2Fe-2S] cluster reacts readily with NO, resulting in the formation of iron-nitrosyl species and a concomitant loss of specific DNA-binding activity.

## Results and Discussion

### NsrR contains an oxygen-insensitive [2Fe-2S] cluster

**Figure 2:**
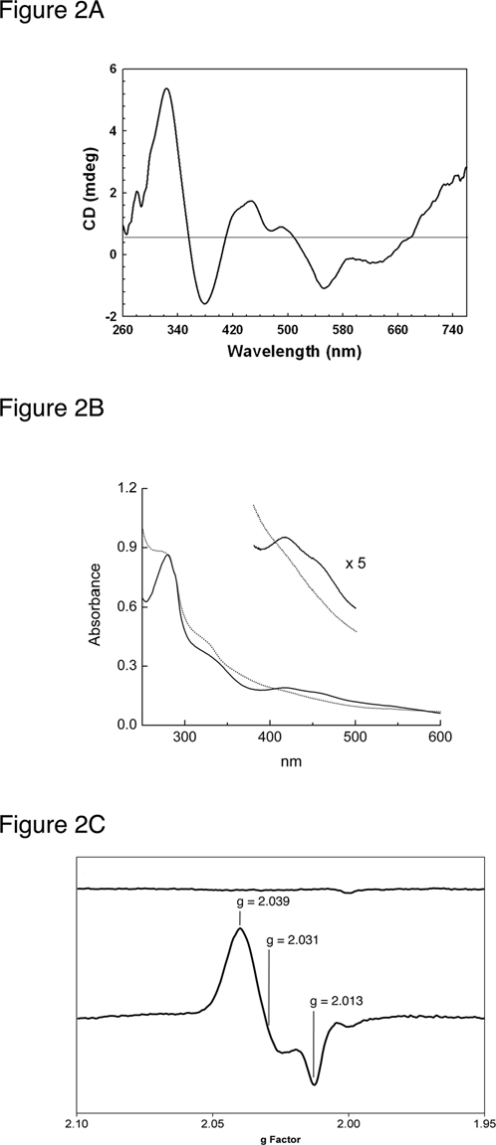
CD, UV-visible and EPR spectroscopy of purified NsrR. (A). The recorded CD spectrum, representing an average of nine individual scans, displays three positive features, λ_max_ 324, 445, 490 nm, together with two negative features, λ_max_ 375 and 550 nm, similar to other [2Fe-2S] cluster containing proteins [20], [21]. The buffer was 75 mM Tris, 425 mM NaCl, 2.5 mM DTT, 2.5% Glycerol, pH 7.5. (B). UV-visible spectra of purified [2Fe-2S] NsrR in 50 mM Tris pH 7.0, 100 mM NaCl buffer or 50 mM Tris pH 7.0, 100 mM NaCl buffer saturated with NO (2 mM), as indicated. UV visible spectroscopy of the purified protein revealed a spectrum characteristic of a [2Fe-2S] cluster containing protein, with major bands at 325 and 420 nm and shoulders present at 460 and 550 nm on the UV visible spectrum [22 Cammack 1980]. The inset spectrum at ×5 magnification shows clearly that the shoulder at 420 nm is lost after exposure to NO (dashed line). (C). X-band EPR spectrum of as isolated NsrR (upper spectrum) and NsrR following exposure to NO (lower spectrum). Measurement conditions were: temperature 10 K; microwave power 2 mW; microwave frequency 9.43755 GHz; modulation amplitude 4 G. NsrR (2.25 uM) was in buffer A and decomposed MAHMA NONOate solutions were prepared in 100 mM Tris-HCl pH 8.0. The purified NsrR protein is EPR silent (top), indicative of an oxidised [2Fe-2S] cluster. Exposure to NO results in a strong signal indicating the formation of a mononuclear dinitrosyl iron complex (bottom, g values are indicated).

Over-expression of *S. coelicolor* NsrR in *E. coli* resulted in cell pellets that were dark brown in color. This color persisted during a two-step purification of NsrR by heparin affinity chromatography and gel filtration on Superdex 75, suggesting the presence of iron in the protein. The CD spectrum of the purified protein displayed bands in the region 260–750 nm with three positive features, l_max_ 324, 445, 490 nm, together with two negative features, l_max_ 375 and 550 nm (Fig. 2A). The bands were of similar energies and the same order of magnitude as those observed for other [2Fe-2S] cluster containing proteins [20], [21], [22], most notably the Rieske protein BphF from *Burkholderia sp* [20]. UV visible spectroscopy (Fig. 2B, solid line) of the purified protein also revealed features characteristic of an [2Fe-2S] protein, with major bands at 325 and 420 nm and shoulders at 460 and 550 nm [23]. Iron and sulfide analysis revealed the presence of 0.9±0.17 irons per sulfide, close to the expected ratio of 1∶1 for a [2Fe-2S] cluster. Furthermore, protein analysis revealed that cluster incorporation was incomplete, with an average of 28% [2Fe-2S] cluster incorporation. This observation is commonly associated with over-expressed iron sulfur proteins. We note that aerobically purified *B. subtilis* NsrR was found to be brownish in color immediately following purification and had an absorption spectrum indicative of an iron sulfur protein. However, the color rapidly faded under aerobic conditions [12] and no further characterization was reported. Surprisingly, the [2Fe-2S] cluster of *S. coelicolor* was stable in the presence of atmospheric oxygen, although dithiothreitol was required to maintain stability. NsrR did not give rise to EPR signals (Fig. 2C, top), consistent with the presence of an oxidized [2Fe-2S]^2+^ cluster. The addition of sodium dithionite did not generate an EPR active species (data not shown). The addition of up to 20 mM sodium dithionite had little effect on the UV-visible absorbance spectrum of NsrR (data not shown), indicating that the cluster is stable in the presence of dithionite. We conclude that the cluster has a reduction potential too low to be effectively reduced by this powerful reductant.

### [2Fe-2S] NsrR reacts readily with NO

Exposure of the protein to NO resulted in an EPR spectrum featuring signals at g = 2.039, 2.0231 and 2.013 (Fig. 2C, bottom). These g-values are characteristic of a S = ½ dinitrosyl iron complex (DNIC) and, importantly, are essentially identical to those previously reported for a DNIC coordinated by cysteine thiolates, Fe(NO)_2_(Cys)_2_ [24], [25], [26]. Similar g-values were also reported following reaction with NO of the iron-sulfur containing proteins aconitase, SoxR and FNR [2], [3], [23]. Quantification of the EPR signal in Fig. 2C revealed that only 8.89% of the iron in the sample was detected in the EPR experiments. This indicates that the majority of the iron was in an EPR-silent form.

Significant changes were also observed in the UV-visible spectrum upon treatment with NO such that features characteristic of the [2Fe-2S] cluster were lost (Fig. 2B, dashed line). Thiol-ligated DNIC species have characteristic absorbance properties: the mononuclear EPR-active DNIC has an absorption maximum at 397 nm while the EPR-silent dinuclear DNIC (in which two iron ions are each ligated by two terminal NO molecules and are bridged by two thiols) gives rise to absorption maxima at 310 and 362 nm [27]. The NO-treated NsrR spectrum (Fig. 2B) is consistent with a mixture of mononuclear and dinuclear thiol-coordinated DNIC species, with a shoulder at ∼310 nm characteristic of the dinuclear DNIC, and broad absorbance out into the visible region consistent with a superposition of mononuclear and dinuclear DNIC absorption envelopes. A proportion of mononuclear DNIC species may be in an EPR-silent state [28]. We note that a similar mixture of DNIC species was observed for NO-treated FNR [2]. Together, these data demonstrate that NO nitrosylates the [2Fe-2S] cluster, resulting in the formation of cysteine thiolate-bound DNIC species. Both the NO treated and untreated NsrR proteins eluted at the same volume on gel filtration (data not shown) suggesting both proteins have the same hydrodynamic radius and that NsrR is not denatured upon treatment with NO.

### Nitric oxide abolishes the DNA binding activity of NsrR

**Figure 3:**
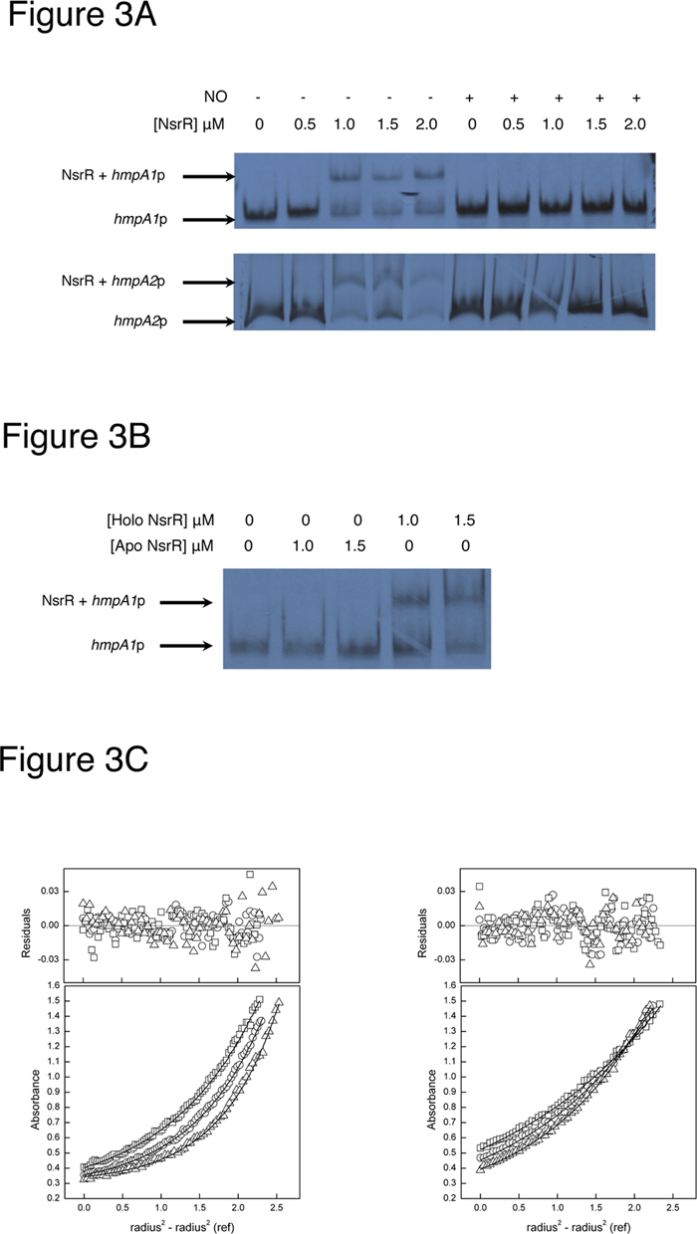
DNA binding assays with the *hmpA1* and *hmpA2* promoters and purified NsrR protein. (A). Bandshift assay using 200 base pair restriction fragments (20 ng per reaction) carrying the *hmpA1* and *hmpA2* promoters, as indicated, and purified [2Fe-2S] NsrR in 50 mM Tris pH 7.0, 100 mM NaCl buffer or 50 mM Tris pH 7.0, 100 mM NaCl buffer saturated with NO (2 mM), as indicated. Binding is abolished by addition of NO saturated buffer to purified NsrR. (B). Bandshift assay using a 200 base pair restriction fragment (20 ng per reaction) carrying the *hmpA1* promoter with either EDTA∶ferricyanide treated apo-NsrR or holo [2Fe-2S] NsrR as indicated in 50 mM Tris pH 7.0, 100 mM NaCl buffer. The apo-form of the protein is unable to bind to the *hmpA1* promoter indicating that the cluster is required for DNA binding activity. (C). Sedimentation equilibrium of oligonucleotides in the presence and absence of purified NsrR. The sedimentation of each sample was monitored at 260 nm and fitted to a single component model as described in Materials and Methods. (Left) Lower panel: absorbance profile of 2 µM *hmpA2* and 10 µM NsrR in 50 mM Tris-HCl pH 7.0, 100 mM NaCl after centrifugation at 16,000 (□), 18,000 (O) and 20,000 (Δ) rpm. Upper panel: Residual profile of the difference between the data and fitted curves. (Right) Lower panel: absorbance profile of 2 µM *hmpA2* in 50 mM Tris-HCl pH 7.0, 100 mM NaCl after centrifugation at 16,000 (□), 18,000 (O) and 20,000 (Δ) rpm. Upper panel: Residual profile of the difference between the data and fitted curves.

To identify potential NsrR target genes, the *S. coelicolor* genome was searched with the NsrR promoter matrix for Bacillales and *Streptomyces* spp (e-cutoff score = 10.7) [16]. A total of 322 genes have potential NsrR binding sites within 70 base pairs of DNA upstream of their translational start codons, including both the *hmpA1* and *hmpA2* genes (Fig. 1B). To investigate whether the purified NsrR protein can bind to these putative sites, bandshift assays were performed using radiolabelled DNA fragments carrying the *hmpA1* and *hmpA2* promoters. The probes were incubated with NsrR diluted in Tris buffer or Tris buffer saturated with NO and the reaction mixtures were separated on a non-denaturing polyacrylamide gel (Fig. 3A). The data clearly demonstrate that NsrR can form a complex with the *hmpA1* and *hmpA2* promoters. NsrR was unable to bind to DNA fragments lacking an NsrR binding site (data not shown). Importantly, DNA binding to the *hmpA1* and *hmpA2* promoters was abolished by NO, demonstrating that NsrR DNA-binding activity is modulated in response to NO. Treatment of the protein with EDTA and ferricyanide [29] resulted in loss of the cluster (as judged from the complete loss of cluster-associated UV visible absorption features, data not shown). Bandshift assays demonstrated that apo-NsrR lacks DNA binding activity (Fig. 3B). A similar loss of DNA binding activity was observed upon addition of the iron chelator bipyridyl to holo NsrR (data not shown). Therefore, we conclude that it is the NO-reactive [2Fe-2S] containing form of NsrR that binds the *hmpA1* and *hmpA2* promoters.

### NsrR binds to DNA as a homodimer

To determine the oligomeric state of [2Fe-2S] NsrR we carried out sedimentation equilibrium experiments at three different speeds using excess NsrR incubated with 30 bp double-stranded oligonucleotide probes carrying either the *hmpA1* or *hmpA2* NsrR binding sites. Oligonucleotides absorb strongly at 260 nm, with an approximate ε_260_ of 370 mM^−1^ cm^−1^, while NsrR has a smaller ε_260_ of ∼9.9 mM^−1^ cm^−1^. This means that even though the NsrR protein is in excess in these experiments, it is the molecular weight of the DNA and DNA-protein complexes that are being measured at 260 nm. A molecular weight of 17.4±0.4 kDa was measured for the *hmpA2* probe, which increased to 47.3±0.9 kDa on incubation with NsrR (Fig. 3B). Since NsrR has a molecular weight of 15.9 kDa, this is consistent with NsrR binding to DNA as a homodimer. The molecular weight of *hmpA1* in the presence of NsrR increased to 45.0±0.6 kDa (data not shown). NsrR was unable to bind to the control probe carrying *nsrR* coding sequence and lacking an NsrR binding site (data not shown) confirming that the interaction is specific.

### The role of NsrR in *Streptomyces*

Members of the genus *Streptomyces* are widespread in soil and are likely exposed to NO produced by microbial denitrification and by the NO synthases of plants and microbes. *S. coelicolor* is resistant to concentrations of up to 6.25 mM of the NO releasing compound S-nitrosoglutathione (GSNO) suggesting it is resistant to NO (data not shown). In similar experiments with *Salmonella* the growth of the wild-type strain was inhibited by 3 mM GSNO [13]. NsrR is the only known NO sensor encoded by the *S. coelicolor* genome suggesting it mediates the response to NO by switching on NsrR dependent genes, including the NO detoxification genes *hmpA1* and *hmpA2*. Of the 322 putative NsrR dependent promoters in *S. coelicolor*, the *hmpA1* and *hmpA2* promoters are ranked 233 and 3, respectively, where a ranking of 1 indicates the closest match to the consensus. Both are bound specifically by NsrR *in vitro* (Fig. 3) suggesting that many, if not all, of the 322 promoters might be NsrR targets. If this is the case then *S. coelicolor* has a large NsrR regulon compared with that of *E. coli* [10] and *N. meningitidis* [14]. Putative targets in *S. coelicolor* include oxidoreductase, hydrolase, ferredoxin, heme oxygenase and DNA repair genes. More unexpected targets include cell wall repair, antibiotic biosynthesis and sporulation genes and several transposases suggesting induction of a global stress response to NO. Furthermore, we have identified 18 promoters that have two NsrR binding sites within 70 bp of the translation start codon. An example of this is the promoter of *SCO0465*, which encodes a non-heme chloroperoxidase, a protein implicated in antibiotic production and the oxidative stress response [30], [31].

### Concluding remarks

The oxygen stable NsrR protein from *S. coelicolor* is an excellent model for studying the NO sensing mechanism of NsrR proteins at the molecular level. We have demonstrated for the first time that NsrR contains a [2Fe-2S] cluster that is required for DNA binding. This cluster is sensitive to NO, reacting to form mononuclear and dinuclear DNIC species. The nitrosylated form of the protein is unable to bind DNA, clearly indicating a possible mechanism by which NsrR regulates gene expression in response to NO. Future work will be aimed at further characterizing NsrR and identifying its target genes *in vivo* in *S. coelicolor*.

## Materials and Methods

### Purification of NsrR

The *nsrR* gene was amplified from *S. coelicolor* genomic DNA using primers nsrRFor (5′-CATATGCGGTTGACGAAGTTCAC) and nsrRRev (5′-TCATCCCGAGGGGCGGTC), cloned into pUC19 for sequencing and then sub-cloned into pET21a to construct plasmid pNsrR. *E. coli* strain BL21 was transformed with pNsrR and single transformant colonies were inoculated into 3×10 ml LB medium and grown overnight with shaking at 37°C. The overnight cultures were used to inoculate 3×1 liter LB in 2.5 liter flasks and cultures were grown with shaking at 37°C to mid log phase. Following induction with 0.1 mM IPTG, and further incubation with shaking at 30°C for 2.5 hours, cells were harvested, resuspended in buffer A (100 mM Tris-HCl pH 8.0, 50 mM NaCl, 5 mM DTT) to a total volume of 40 ml and passed three times through a French pressure cell at 1000 Psi. The crude lysate was then centrifuged in a Sorvall SS-34 rotor at 18,000 rpm for 45 minutes to generate a clarified NsrR-containing extract. The clarified extracts were applied to a 5 ml HiTrap Heparin HP column (GE Healthcare) at room temperature and the column was washed in buffer A at 5 ml/min until the UV trace became stable. Proteins were eluted using a gradient of buffer B (100 mM Tris-HCl pH 8.0, 1 M NaCl, 5 mM DTT) equivalent to 20 column volumes. Fractions containing NsrR were identified by their dark brown color and by SDS-PAGE. The most concentrated fractions were applied to a Superdex 75 26/60 column (GE Healthcare) using a 2 ml superloop at a flowrate of 1.5 ml/min. Fractions (1.5 ml) were collected and analysed by SDS-PAGE; those containing pure NsrR were pooled and stored at −20°C in buffer B+5% glycerol until use. Apo-NsrR was prepared by incubating the purified protein with EDTA and ferricyanide in a molar ratio of protein∶EDTA∶ferricyanide (1∶50∶20) at 25°C for 20 minutes [29] before buffer exchanging the protein with buffer A. Loss of cluster was verified by measuring the UV-visible absorbance spectrum of the treated protein, as described below. Bipyridyl treatment of purified NsrR (10 µM) was carried out at a final bipyridyl concentration of 1 mM in DNA binding buffer (10 mM Tris-HCl, pH 7.5, 60 mM KCl) on ice for 30 minutes. The protein was then used directly in bandshift reactions (see below).

### Near UV- CD, UV visible CD, UV-visible absorbance and EPR spectroscopy

NsrR was exchanged into buffer C (50 mM Tris pH 7.0, 100 mM NaCl) to remove excess DTT. A Jasco J-810 spectrophotometer, scanning at 200 nm min^−1^, was used to record the CD spectrum of NsrR using a standard 1 cm cuvette. The UV-visible absorbance spectrum of a 200 µL sample of NsrR was measured between 250 and 600 nm on a Hitachi V-3310 dual beam spectrophotometer. After recording the spectrum of the oxidised protein 10 µL of a 2 mM NO stock solution was added to give a final concentration of 100 µM NO in the cuvette. The spectrum of the NO incubated NsrR was immediately recorded and corrected. Absorbance traces were normalized to account for the small increase in volume after addition of NO.

X-band EPR spectra were recorded on a Bruker ELEXSYS 500 fitted with a Bruker Super-High-Q Cavity: ER 4122SHQE and the temperature was controlled using an Oxford Instruments ESR-9 flow cryostat. Spectra were recorded at 10 K, with a frequency of 9.437 GHz, power of 2 mW. 150 µL of NsrR (2.25 µM) was either mixed with 50 µL of NO saturated buffer C or with 50 µL of decomposed 100 mM MAHMA-NONOate to ensure an excess of NO. In samples where NO was omitted, 50 µL of buffer C was added as a control. Spin quantification was carried out using the method of Aasa and Vänngård as described previously, using 1 mM of aqueous Cu(II)(H_2_O)_6_ as a concentration standard [32], [33].

### Bandshift assays

DNA fragments carrying the *hmpA1* and *hmpA2* promoters were PCR amplified using *S. coelicolor* genomic DNA with primers phmpA1F (5′-GACGGACCGCCCCTCGGGA), phmpa1R (5′-GCGATGTCACCGATGGCCGCT), phmpA2F (5′-TCCGGCCGCTGTCCGGTCT) and phmpa2R (5′-GATCGTGCCGAGCGAGGCT) and cloned into pUC19 for sequencing. The fragments were excised from pUC19 with *EcoR*I and *Hin*dIII, gel purified twice and ∼1 µg of each fragment was radiolabelled using Klenow and a mixture of [α^35^S] dATP (Perkin Elmer) and cold dCTP, dGTP and dTTP for 15 minutes at room temperature. Radio-labelled probes were purified on Qiagen nucleotide removal columns and stored at −20°C. Purified NsrR protein was diluted in 50 mM Tris-HCl (pH 7.0) buffer or 50 mM Tris-HCl (pH 7.0) buffer saturated with NO gas (2 mM NO solution), to a final concentration of 500 nM. Bandshift reactions (10 µL) containing ∼20 ng radiolabelled probe, 10 mM Tris-HCl, pH 7.5, 60 mM KCl were incubated with and without NsrR protein on ice for 2 minutes. Loading dye (2 µL) was added and reaction mixtures were separated on 7.5% polyacrylamide gels in 1× Tris-Borate-EDTA buffer in a Mini Protean III system (BioRad) at 100 volts for 1 hour and then dried and exposed to XRay film overnight.

### Analytical ultracentrifugation

Oligonucleotides carrying either the *hmpA1* binding site (*hmpA1* For 5′-CTAAAACACGAATATCATCTACCAATTAAG and *hmpA1* Rev 5′-CTTAATTGGTAGATGATATTCGTGTTTTAG), *hmpA2* binding site (*hmpA2* For 5′-GGAAAACAAGCATCTGAGATCCCAGTTCGG and *hmpA2* Rev 5′- CCGAACTGGGATCTCAGATGCTTGTTTTCC) or no NsrR binding site (Control For 5′-CCGCCTGCAGGCCCTGGGTGTG and Control Rev 5′- CACACCCAGGGCCTGCAGGCGG) were annealed by heating to 94°C for 5 minutes and cooling to 55°C for 5 minutes. The double stranded oligonucleotides were then used as probes. The calculated molecular weights of the double stranded *hmpA1*, *hmpA2* and Control oligonucleotides were 18.4 kDa, 18.4 kDa and 13.5 kDa respectively, and the partial specific volume (v-bar) of the oligonucleotide was estimated as 0.55 mL g^−1^ [33]. The molecular weight of the monomeric NsrR polypeptide was calculated as 15.9 kDa and v-bar as 0.74 mL g^−1^. Sedimentation equilibrium experiments were performed in a Beckman Optima XL-I analytical ultracentrifuge equipped with absorbance optics and an An50Ti rotor. Samples containing 2 µM oligonucleotide, 10 µM monomeric NsrR in 50 mM Tris pH 8.0, 100 mM NaCl, 1 mM dithiothreitol were loaded into charcoal-filled Epon double sector cells fitted with quartz windows. 110 µL of sample was loaded into the sample sector and 120 µL of buffer was loaded into the reference sector. Samples were centrifuged at speeds of 16,000, 18,000 and 20,000 rpm and the absorbance was recorded at 260 nm. Data analysis was executed using Ultrascan II [34] where 3 scans at 3 different speeds were simultaneously fitted to a one-component model. Fitting of a single scan at 3 speeds was used to measure the standard error of the obtained data.

## References

[1] Lane N (2007). Climate change: What's in the rising tide?. Nature.

[2] Cruz-Ramos H, Crack J, Wu G, Hughes MN, Scott C (2002). NO sensing by FNR: regulation of the *Escherichia coli* NO-detoxifying flavohaemoglobin, Hmp.. EMBO J.

[3] Ding H, Demple B (2000). Direct nitric oxide signal transduction via nitrosylation of iron-sulfur centers in the SoxR transcription activator.. Proc Natl Acad Sci U S A.

[4] D'Autreaux B, Tucker NP, Dixon R, Spiro S (2005). A non-haem iron centre in the transcription factor NorR senses nitric oxide.. Nature.

[5] Gardner AM, Helmick RA, Gardner PR (2002). Flavorubredoxin, an inducible catalyst for nitric oxide reduction and detoxification in *Escherichia coli*.. J Biol Chem.

[6] Hutchings MI, Mandhana N, Spiro S (2002). The NorR Protein of *Escherichia coli* Activates Expression of the Flavorubredoxin Gene *norV* in Response to Reactive Nitrogen Species.. J Bacteriol.

[7] Tucker NP, D'Autreaux B, Studholme DJ, Spiro S, Dixon R (2004). DNA binding activity of the *Escherichia coli* nitric oxide sensor NorR suggests a conserved target sequence in diverse proteobacteria.. J Bacteriol.

[8] Beaumont HJ, Lens SI, Reijnders WN, Westerhoff HV, van Spanning RJ (2004). Expression of nitrite reductase in Nitrosomonas europaea involves NsrR, a novel nitrite-sensitive transcription repressor.. Mol Microbiol.

[9] Bodenmiller DM, Spiro S (2006). The *yjeB* (*nsrR*) gene of Escherichia coli encodes a nitric oxide-sensitive transcriptional regulator.. J Bacteriol.

[10] Filenko N, Spiro S, Browning DF, Squires D, Overton TW (2007). The NsrR regulon of *Escherichia coli* K-12 includes genes encoding the hybrid cluster protein and the periplasmic, respiratory nitrite reductase.. J Bacteriol.

[11] Poole RK (2005). Nitric oxide and nitrosative stress tolerance in bacteria.. Biochem Soc Trans.

[12] Nakano MM, Geng H, Nakano S, Kobayashi K (2006). The nitric oxide-responsive regulator NsrR controls ResDE-dependent gene expression.. J Bacteriol.

[13] Gilberthorpe N, Poole RK (2007). NsrR: a key regulator circumventing *Salmonella enterica* serovar Typhimurium oxidative and nitrosative stress in vitro and in IFN-c-stimulated J774.2 macrophages.. Microbiology.

[14] Rock JD, Thomson MJ, Read RC, Moir JW (2007). Regulation of denitrification genes in *Neisseria meningitidis* by nitric oxide and the repressor NsrR.. J Bacteriol.

[15] Overton TW, Whitehead R, Li W, Snyder LA, Saunders NJ (2006). Coordinated regulation of the Neisseria gonorrhoeae-truncated denitrification pathway by the nitric oxide-sensitive repressor, NsrR, and nitrite-insensitive NarQ-NarP.. J Biol Chem.

[16] Rodionov DA, Dubchak IL, Arkin AP, Alm EJ, Gelfand MS (2005). Dissimilatory metabolism of nitrogen oxides in bacteria: comparative reconstruction of transcriptional networks.. PLoS Comput Biol.

[17] Schwartz CJ, Giel JL, Patschkowski T, Luther C, Ruzicka FJ (2001). IscR, an Fe-S cluster-containing transcription factor, represses expression of *Escherichia coli* genes encoding Fe-S cluster assembly proteins.. Proc Natl Acad Sci U S A.

[18] Todd J, Wexler M, Sawers G, Yeoman KH, Poole P (2002). RirA, an iron-responsive regulator in the symbiotic bacterium Rhizobium leguminosarum.. Microbiology.

[19] Challis GL, Hopwood DA (2003). Synergy and contingency as driving forces for the evolution of multiple secondary metabolite production by *Streptomyces* species.. Proc Natl Acad Sci USA.

[20] Couture MM, Colbert CL, Babini E, Rosell FI, Mauk AG (2001). Characterization of BphF, a Rieske-type ferredoxin with a low reduction potential.. Biochemistry.

[21] Kimura S, Kikuchi A, Senda T, Shiro Y, Fukuda M (2005). Tolerance of the Rieske-type [2Fe-2S] cluster in recombinant ferredoxin BphA3 from Pseudomonas sp. KKS102 to histidine ligand mutations.. Biochem J.

[22] Stephens PJ, Thomson AJ, Dunn JB, Keiderling TA, Rawlings J (1978). Circular dichroism and magnetic circular dichroism of iron-sulfur proteins.. Biochemistry.

[23] Cammack R (1980). New developments in iron-sulphur proteins.. Nature.

[24] Kennedy MC, Antholine WE, Beinert H (1997). An EPR investigation of the products of the reaction of cytosolic and mitochondrial aconitases with nitric oxide.. J Biol Chem.

[25] Vanin AF, Serezhenkov VA, Mikoyan VD, Genkin MV (1998). The 2.03 signal as an indicator of dinitrosyl-iron complexes with thiol-containing ligands.. Nitric Oxide.

[26] Pierce BS, Gardner JD, Bailey LJ, Brunold TC, Fox BG (2008). Characterization of the nitrosyl adduct of substrate-bound mouse cysteine dioxygenase by electron paramagnetic resonance: electronic structure of the active site and mechanistic implications.. Biochemistry.

[27] Costanzo S, Menage S, Purrello R, Bonomo RP, Fontecave M (2001). Re-examination of the formation of dinitrosyl – iron complexes during reaction of S -nitrosothiols with Fe(II).. Inorganica Chimica Acta.

[28] D'Autréaux B, Horner O, Oddou JL, Jeandey C, Gambarelli S (2004). Spectroscopic description of the two nitrosyl-iron complexes responsible for fur inhibition by nitric oxide.. J Am Chem Soc.

[29] Alam MS, Garg SK, Agrawal P (2007). Molecular function of WhiB4/Rv3681c of *Mycobacterium tuberculosis* H37Rv: a [4Fe-4S] cluster co-ordinating protein disulphide reductase.. Mol Microbiol.

[30] Wiesner W, van Pée KH, Lingens F (1998). Purification and characterization of a novel bacterial non-heme chloroperoxidase from *Pseudomonas pyrrocinia*.. J Biol Chem.

[31] De Mot R, Schoofs G, Nagy I (2007). Proteome analysis of *Streptomyces coelicolor* mutants affected in the proteasome system reveals changes in stress-responsive proteins.. Arch Microbiol.

[32] Aasa R, Vänngård T (1975). EPR signal intensity and powder lineshapes: a reexamination.. J Magnet Reson.

[33] Tucker NP, D'Autréaux B, Yousafzai FK, Fairhurst SA, Spiro S (2008). Analysis of the nitric oxide-sensing non-heme iron center in *the NorR regulatory* protein.. J Biol Chem.

[34] Cantor CR, Schimmel PR (1980). Biophysical Chemistry, 2nd ed., Vol. II.

[35] Demeler B, Scott DJ, Harding SE, Rowe AJ (2005). Analytical ultracentrifugation.

